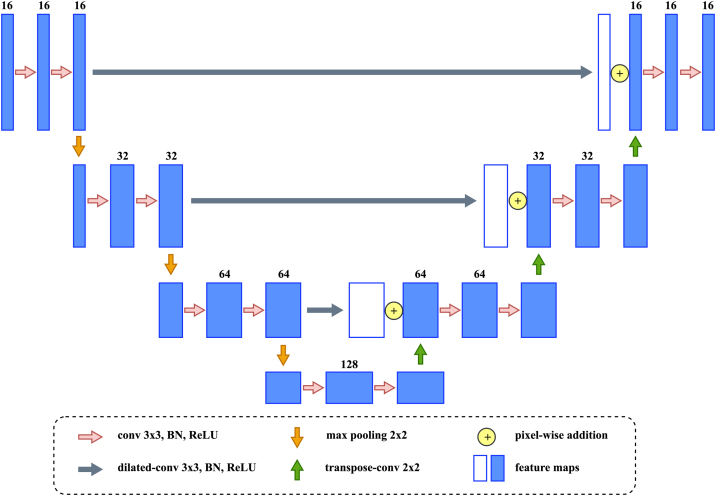# Fully automatic framework for comprehensive coronary artery calcium scores analysis on non-contrast cardiac-gated CT scan: Total and vessel-specific quantifications

**DOI:** 10.1016/j.ejrad.2020.109420

**Published:** 2021-01

**Authors:** Nan Zhang, Guang Yang, Weiwei Zhang, Wenjing Wang, Zhen Zhou, Heye Zhang, Lei Xu, Yundai Chen

**Affiliations:** a Department of Radiology, Beijing Anzhen Hospital, Capital Medical University, 2^nd^ Anzhen Road, Chaoyang District, Beijing, China; b Cardiovascular Research Centre, Royal Brompton Hospital, SW3 6NP, London, UK; c National Heart and Lung Institute, Imperial College London, London, SW7 2AZ, UK; d School of Biomedical Engineering, Sun Yat-Sen University, China; e Department of Cardiology, Chinese PLA General Hospital, Beijing, China

**Keywords:** CAC, Coronary artery calcium, CACS, Coronary artery calcium scores, ICC, Inter-class correlation coefficient, MACE, Major adverse cardiovascular events, CAC-DRS, CAC data and reporting system, CHD, Coronary heart disease, CVD, Cardiovascular disease, CT, Computed Tomography, HR, Heart rate, LM, Left main artery, LAD, Left ascending artery, LCX, Left circumflex artery, RCA, Right coronary artery, MVSC, Multiview shape constraint, AS, Agatston score, VS, Calcium volume score, MS, Calcium mass score, #CV, Number of calcified vessels, Calcium, Coronary artery disease, Deep learning, Tomography, X-ray computed

## Abstract

**Objectives:**

To develop a fully automatic multiview shape constraint framework for comprehensive coronary artery calcium scores (CACS) quantification via deep learning on nonenhanced cardiac CT images.

**Methods:**

In this retrospective single-centre study, a multi-task deep learning framework was proposed to detect and quantify coronary artery calcification from CT images collected between October 2018 and March 2019. A total of 232 non-contrast cardiac-gated CT scans were retrieved and studied (80 % for model training and 20 % for testing). CACS results of testing datasets (n = 46), including Agatston score, calcium volume score, calcium mass score, were calculated fully automatically and manually at total and vessel-specific levels, respectively.

**Results:**

No significant differences were found in CACS quantification obtained using automatic or manual methods at total and vessel-specific levels (Agatston score: automatic 535.3 vs. manual 542.0, P = 0.993; calcium volume score: automatic 454.2 vs. manual 460.6, P = 0.990; calcium mass score: automatic 128.9 vs. manual 129.5, P = 0.992). Compared to the ground truth, the number of calcified vessels can be accurate recognized automatically (total: automatic 107 vs. manual 102, P = 0.125; left main artery: automatic 15 vs. manual 14, P = 1.000 ; left ascending artery: automatic 37 vs. manual 37, P = 1.000; left circumflex artery: automatic 22 vs. manual 20, P = 0.625; right coronary artery: automatic 33 vs. manual 31, P = 0.500). At the patient’s level, there was no statistic difference existed in the classification of Agatston scoring (P = 0.317) and the number of calcified vessels (P = 0.102) between the automatic and manual results.

**Conclusions:**

The proposed framework can achieve reliable and comprehensive quantification for the CACS, including the calcified extent and distribution indicators at both total and vessel-specific levels.

## 1 Introduction

Coronary artery calcium (CAC) scores (CACS) are widely used quantification indices to describe the extent of coronary arteriosclerosis in daily clinical routine. In the past decades, CACS have been proven as an independent risk factor for predicting major adverse cardiovascular events (MACE) and death in several particular patient cohorts. The CACS have therefore been integrated into several clinical application guidelines and expert consensus [[1], [2], [3], [4], [5], [6]]. Recently, in order to standardize the clinical procedure, new CAC data and reporting system (CAC-DRS) was released [7]. According to the CAC-DRS, calcification extent and distribution should be reported using the Agatston score or via a visual estimation method depending on the CT scanning protocol.

The Agatston score is a widely used and recommended indicator to evaluate the calcification extent [7]. In addition to the Agatston score, there are some other CAC characteristics, such as CAC volume, density and mass can be used to describe the calcification extent. However, the prognostic power of these CAC characteristics has remained controversial [8,9].

In recent studies, distribution of the CAC has been reported to predict coronary heart disease (CHD), cardiovascular disease (CVD) and all-cause death independently, and it has also been used to provide additional value for the improvement of risk stratification rather than using the Agatston score alone [[10], [11], [12]]. In the CAC-DRS, it has recommended that the vessel-specific CACS and the number of calcified vessels should also be reported to describe the distribution of the CAC [7].

Different studies of automatic CACS evaluation methods have been published to tackle this repeated, tedious and time-consuming manual procedure [[13], [14], [15], [16], [17], [18], [19], [20], [21], [22]]. CACS results can be reported on patient, vessel or lesion levels automatically from non-contrast CT data using these methods. However, false positive and false negative result happened, due to the misclassification of the aortic calcification close to the coronary ostia and the calcification at the left main or proximal left anterior descending artery [23]. Currently, only limited previous studies have investigated the automated calculation of the CACS for the left main artery separately, which could be considered as an independent risk factor for cardiovascular and total mortality in asymptomatic adults [24]. Moreover, the number of calcified vessels indicating the calcification distribution, which is recommended by the CAC-DRS, has yet been investigated in previous studies.

According to the recommendation of the CAC-DRS, we propose a fully automatic framework for CACS evaluation, including comprehensive calcification extent and distribution assessment, to provide accurate information and indicators for the improvement of risk discrimination and classification.

## 2 Materials and methods

This retrospective study was approved by our institutional review board in accordance with local ethics procedures.

### 2.1 Study participants

**Fig. 1:**
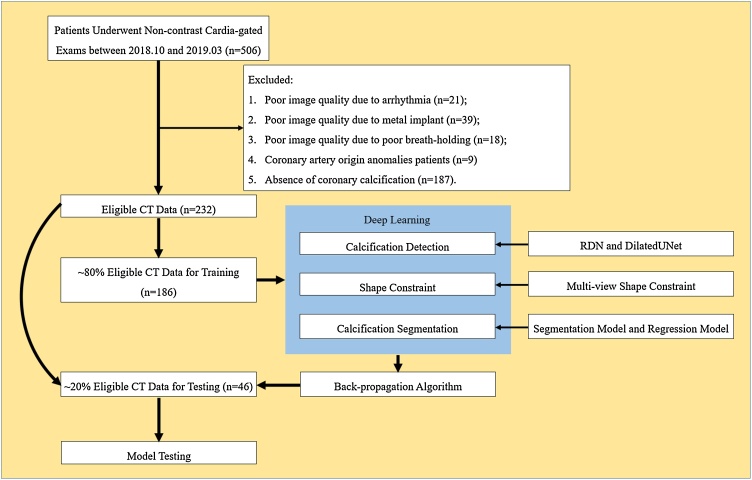
Flowchart showing the patients (n = 232) received non-contrast cardiac-gated CT scans (selected from 506 patients scanned between October 2018 and March 2019). During model training 186 patients were used, and a supervised deep learning framework was developed to detect and localize the coronary artery calcification. Predictive performance of the deep learning was assessed using an testing scheme on 46 participants.

In total, we retrospectively retrieved non-contrast cardiac-gated CT scans from consecutively enrolled 506 patients suspected with CHD for the CACS assessment (scanned between October 2018 and March 2019). In our study, 78 patients were excluded because of poor image quality, e.g., due to arrhythmia (n = 21), the metal implant (n = 39) and poor breath-holding (n = 18). Besides, coronary artery origin anomalies (n = 9) and coronary calcification absence (n = 187) cases were also excluded. Finally, 232 non-contrast cardiac-gated CT scans were enrolled, in which 80 % (n = 186) were used for training, and 20 % (n = 46) were used for independent testing (Fig. 1).

### 2.2 Imaging protocol

CT scans were performed using a second-generation 128-slice dual-source computed tomography system (SOMATOM Definition Flash, Siemens Healthcare, Forchheim, Germany). All scans were performed in a craniocaudal direction with a standard prospective cardiac-gated protocol [25]. Exposure interval was chosen depending on the heart rate (HR): 30–40 % RR interval for patients with an HR ≥ 70 bpm, 70–80 % RR interval for patients with an HR < 70 bpm. Acquisition range covered 1 cm below the carina to cardiac apex. Scanning parameters were defined as follows: slice collimation of 2 × 64 × 0.6 mm, the field of view of 220 × 220 mm, gantry rotation time of 280 ms, tube voltage of 120 kV, and the tube current was adjusted as a function of patient size. CT images were reconstructed at axial, sagittal, and coronal views with a conventional filtered back projection algorithm (B26f Medium Smooth ASA) at 3 mm slice thickness with a 3 mm increment.

### 2.3 Radiologists readout

#### 2.3.1 CAC detection and delineation

A radiologist (N.Z., with seven years of experience in cardiovascular CT image) manually performed CAC delineation on an axial view of the CT images and also referred to complementary information from sagittal and coronal views, using 130Hu as a threshold to identify the calcification [26]. All manual segmentation results were reviewed by another expert radiologist (L.X., with ten years of experience in cardiovascular CT image). In cases of disagreement, the consensus between two radiologists is used as gound-truth CAC label.

#### 2.3.2 CACS evaluation

CACS, including Agatston score (AS), calcium volume score (VS) and calcium mass score (MS), were estimated based on manual mask of coronary calcification, which were obtained by Matlab according to clinical calcium scoring standards [27]. The result of CACS was respectively recorded at total and vessel-specific levels, i.e., left main artery (LM), left ascending artery (LAD), left circumflex artery (LCX) and right coronary artery (RCA).

#### 2.3.3 CAC-DRS classification

At the patient’s level, according to CAC-DRS recommendation [7], CACS were recorded as 4 grades with total AS result: CAC-DRS A0 (AS = 0), CAC-DRS A1 (AS 1–99), CAC-DRS A2 (AS 100–299), CAC-DRS A3 (AS > 300). The number of calcified vessels was recorded as N0–N4 for each patient.

### 2.4 Deep learning model

**Fig. 2:**
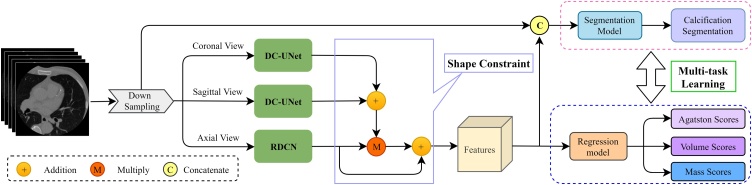
Overview of the proposed MVSC framework.

The proposed MultiView Shape Constraint (MVSC) framework leveraged the multiview learning to mimic the reporting clinician's routine inspection procedure of CAC that focuses on the axial planar information of 3D CT scans but also collects auxiliary information from coronal and sagittal views. Then, a segmentation model and a regression model were combined to disentangle the multiview features for comprehensive calcification analysis. Calcification masks were obtained via the segmentation model. And CACS, including AS, VS, MS, were estimated via the regression model, at total and vessel-specific levels, respectively. The overview of our MVSC is shown in Fig. 2. Details of the MVSC framework was provided in the supplementary material.

### 2.5 Statistical analysis

Statistical analyses were performed using SPSS 23.0 (SPSS, Inc., Chicago, IL). For demographics information, independent t-tests and Mann-Whitney U tests were used to compare differences between two groups of continuous and dichotomous variables, respectively. For the testing datasets, differences between CACS results obtained automatically and manually, including AS, VS and MS, were compared using paired t-testing at total and vessel-specific levels, respectively. Besides, differences in the number of calcified vessels calculated automatically and manually at total and vessel-specific levels were compared using McNemar tests. With manually assessed result as the ground truth, the reliability of our fully automatic framework was evaluated using the inter-class correlation coefficient (ICC) and Bland-Altman analysis. At the patient’s level, differences of CAC-DRS classification calculated automatically and manually were compared by using paired Wilcoxon signed-rank test. In addition, a two-sided P < 0.05 was considered to be statistically significant.

## 3 Results

### 3.1 Study population characteristics

Based on gender, age, weight, height, BMI and other coronary risk factors, patients with risk of coronary artery disease received non-contrast cardiac-gated CT scans were selected as training and testing participants. There were no significant differences found in population characteristics between patients in training and testing datasets except the age (training datasets: 55.7 ± 13.2 vs. testing datasets: 61.9 ± 9.9, P = 0.003). (Table 1)

**Table 1:** Demographics of the patients.

Characteristics		Training datasets (n = 186)	Testing datasets (n = 46)	P
Male		162 (87.1 %)	40 (87.0 %)	0.901
Age (years)		55.7 ± 13.2	61.9 ± 9.9	0.003
Weight (kg)		75.5 ± 16.2	70.9 ± 8.5	0.068
Height (cm)		166.8 ± 16.8	168.7 ± 7.5	0.507
BMI (kg/m^2^)		27.6 ± 16.9	24.9 ± 2.3	0.324
Coronary risk factors	Hypertension	104 (55.9 %)	26 (56.5 %)	1.000
	Diabetes	66 (35.5 %)	23 (50.0 %)	0.079
	Smoking	108 (58.1 %)	30 (65.2 %)	0.420
	Dyslipidemia	57 (30.6 %)	13 (28.3 %)	0.721
	Family history	7 (3.8 %)	2 (4.3 %)	0.865

### 3.2 Testing on CACS

**Fig. 3:**
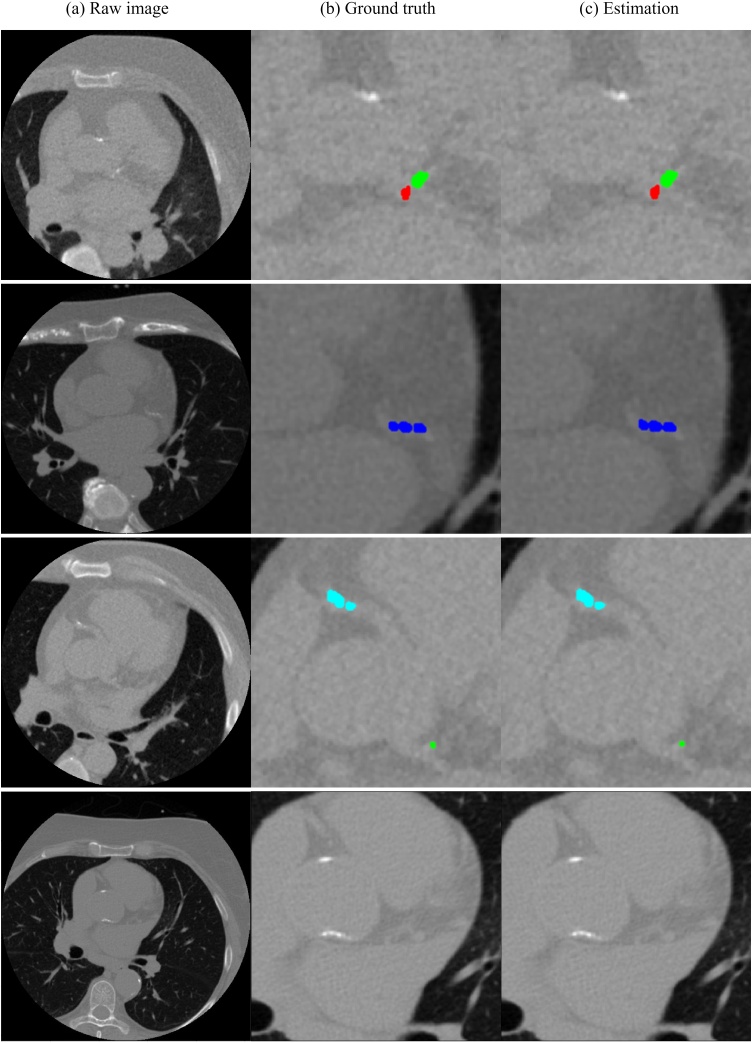
Visualization of the segmentation results for three example cases (each row represents an example case). Column (a): Raw image; Column (b): Ground truth and Column (c) Estimation. Zoomed-in views show the estimated calcification vs. the ground truth (Red: LM calcification, Green: LAD calcification, Blue: LCX calcification, Cyan: RCA calcification) (F1-score: LM: 0.965, LAD: 0.969, LCX: 0.958, RCA: 0.972). The last row showed that our MVSC could effectively exclude aorta calcification.

Compared to the ground truth, our fully automatic framework can accurately estimate the CACS from non-contrast cardiac-gated CT scans at both total and vessel-specific levels (Fig. 3).

There were no significant differences in the quantification of the CACS, including AS, VS and MS, between the results obtained automatically and manually at total or vessel-specific levels (Table 2). For the AS assessment, the result of the test group was 535.3 at the total level measured automatically, which was almost equal to the ground truth (compared to the result obtained manually: 542.0, P = 0.993). Automatic result was also close to the ground truth at the vessel-specific level for the AS (LM: automatic 32.9 vs. manual 32.4, P = 0.986; LAD: automatic 318.7 vs. manual 326.4, P = 0.989; LCX: automatic 51.6 vs. manual 51.4, P = 0.966; RCA: automatic 127.4 vs. manual 131.8, P = 0.983).

**Table 2:** Quantified CACS results of the testing at total and vessel-specific levels.

		AS			VS (mm^3^)			MS (mg/cm^3^)		
		Automatic	Manual	P	Automatic	Manual	P	Automatic	Manual	P
Total		535.3	542.0	0.993	454.2	460.6	0.990	128.9	129.5	0.992
Vessel-specific	LM	32.9	32.4	0.986	21.9	21.2	0.987	6.1	5.9	0.988
	LAD	318.7	326.4	0.989	270.2	273.4	0.989	78.1	78.3	0.991
	LCX	51.9	51.4	0.968	40.8	41.2	0.963	13.6	13.8	0.966
	RCA	127.4	131.8	0.983	121.5	124.8	0.974	31.1	31.5	0.980

### 3.3 Testing on the number of calcified vessels

As shown in Table 3, compared to the manually defined ground truth, the number of calcified vessels can be accurately recognized using our automatic framework (total: automatic 107 vs. manual 102, P = 0.125; LM: automatic 15 vs. manual 14, P = 1.000 ; LAD: automatic 37 vs. manual 37, P = 1.000; LCX: automatic 22 vs. manual 20, P = 0.625; RCA: automatic 33 vs. manual 31, P = 0.500).

**Table 3:** Comparison of the number of calcified vessels using testing datasets.

		Automatic	Manual	P
Total		107	102	0.125
Vessel-specific	LM	15	14	1.000
	LAD	37	37	1.000
	LCX	22	20	0.625
	RCA	33	31	0.500

### 3.4 Consistency analysis

**Fig. 4:**
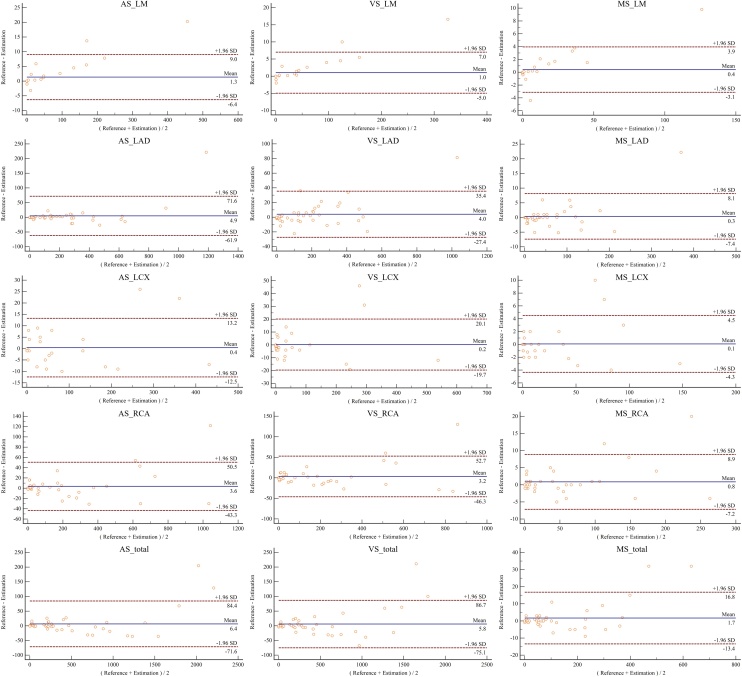
Bland-Altman analysis of the CACS quantification between automatic and manual results at total and vessel-specific levels.

At the total level, we obtained consistently high ICC values for the CACS quantification and also for the number of calcified vessels assessment between the automatic and manual results (AS: 0.988, P = 0.998; VS: 0.985, P = 0.998; MS: 0.987, P = 0.998; the number of calcified vessels: 0.997, P = 0.998). At the vessel-specific level, we also obtained consistently high ICC values for the CACS assessment between automatic and manual results, with the lowest ICC at the LCX (Table 4). Fig. 4 showed good consistency between automatic and manual results at both total and vessel-specific levels for the AS, VS and MS using Bland-Altman analysis. As shown in Fig. 4, larger CACS resulted in larger bias between estimation and ground truth. Our MSVC applied MAE as the loss function of the regression model, which was relatively insensitive to outliers.

**Table 4:** Consistency analysis using ICC for the CACS at total and vessel-specific levels (# CV: the number of calcified vessels).

		AS	P	VS	P	MS	P	# CV	P
Total		0.988	0.998	0.985	0.998	0.987	0.998	0.997	0.998
Vessel-specific	LM	0.980	0.993	0.977	0.991	0.981	0.994	1.000	1.000
	LAD	0.986	0.997	0.984	0.997	0.986	0.998	1.000	1.000
	LCX	0.965	0.990	0.959	0.989	0.958	0.985	0.995	0.997
	RCA	0.978	0.996	0.974	0.993	0.976	0.993	0.996	0.998

Consequently, the model acquired higher accuracy on small CACS but lower accuracy on large CACS. In each Bland-Altman plot, at most two samples (4.3 %, 2/46) located outside the 95 % limits of agreement (95 % LoA). 95 % LoA for AS assessment on the total level was -62.4∼69, which can be accepted in clinical practice by using CAC-DRS categories of hundreds AS scales.

### 3.5 CAC-DRS classification

We have stratified the estimated patients based on the CAC-DRS recommendation. As shown in Table 5, one patient in A0 is incorrectly classified as A1 (P = 0.317). At the patient’s level, there was no statistic difference existed in the classification of the number of calcified vessels between the automatic and manual results (N0: automatic 8 vs. manual 9; N1: automatic 5 vs. manual 6; N2: automatic 8 vs. manual 7; N3: automatic 14 vs. manual 14; N4: automatic 11 vs. manual 10, P = 0.102).

**Table 5:** Comparison of CAC-DRS classification using testing datasets.

AS			#CV		
	Automatic	Manual		Automatic	Manual
A0	8	9	N0	8	9
A1	8	7	N1	5	6
A2	11	11	N2	8	7
A3	19	19	N3	14	14
			N4	11	10
P	0.317		P	0.102	

### 3.6 Computational time

The training time of our deep learning method was ∼33 h using all the 186 datasets. In the test phase, the computational time is ∼1.5 s for one 3D CT scan.

## 4 Discussion

In our study, we have developed and verified a new automatic framework for comprehensive CACS quantification and analysis on non-contrast cardiac-gated CT scans at total and vessel-specific levels. Excellent consistency has been achieved between manual ground truth and results obtained using our automatic method. With the proposed innovative deep learning based algorithm, CAC can be automatically identified and quantified accurately and efficiently (e.g., ∼1.5 s per 3D CT scan).

Accurate detection of the CAC is a difficult task because of the false positives from the calcification at aortic root and annulus. Moreover, CAC at the left main artery could be misclassified as the aortic calcification to result in false negatives [23]. The misclassification would reduce the accuracy of CACS measurement at LM and RCA, because of the adjacent relationship between the two arteries and aortic root. In this study, we proposed an MVSC framework to combine the vessel segmentation and the calcification segmentation models for better detection and quantification for the CAC. Using manual results as standard, automatic CACS results of LM and RCA was accurate without obvious over- or under-estimation (LM: AS: 32.9 (Automatic) vs. 32.4 (Manual), P = 0.986; RCA: AS: 127.4 (Automatic) vs. 131.8 (Manual), P = 0.983). Furthermore, number of calcified vessels was evaluated accurately automatically (LM: 15 (Automatic) vs. 14 (Manual), P = 1.000; RCA: 33 (Automatic) vs. 31 (Manual), P = 0.500). Calcification was overestimated in 1 LM and 2 RCA vessels because of calcification existed in the aortic sinus, which was tightly attached with the vessels.

CACS at the left main coronary artery is independently associated with a 20–30 % greater hazard for cardiovascular and total mortality in asymptomatic adults [24]. A previous study has also demonstrated that compared to the circumflex artery, right coronary artery and left anterior descending artery, Agatston score at left main coronary artery is associated with a higher relative risk for death in asymptomatic adults [27]. In addition, among all the CACS at vessel-specific level, adjusted for traditional clinical risk factors, CACS at the left main coronary artery and left anterior descending artery have a significant association with the mortality [28]. However, using the traditional Agatston score, CACS of the left main coronary artery can weight equally to the CACS at distal vessels and segments [7]. Thus, CAC-DRS has recommended reporting the CACS at both total and vessel-specific levels. In this study, the proposed MVSC framework can automatically and accurately quantify the CACS at both total and vessel-specific levels, especially for the CACS evaluation at the left main coronary artery. Using our MVSC framework, we can envisage an improvement of the risk stratification and personalized pharmacotherapy for asymptomatic patients.

Besides CAC at left main coronary artery, distributed information of the CAC, for example, the number of calcified vessels, plaques and CACS present at proximal arteries should also be evaluated to provide potential indicators for better risk discrimination and reclassification, which has not been done in previous studies [24]. In this study, using our proposed automatic framework, there were no significant differences found on the number of calcified vessels at both total and vessel-specified levels compared to the results obtained manually. Thus, the proposed framework can not only accurately evaluate the CACS, but can also estimate the number of calcified vessels as recommended by the CAC-DRS [7].

An 18-segment model proposed by the Society of Cardiovascular Computed Tomography was used to define the coronary arterial segments [21]. CACS result on the segmental level can be used to provide distributed information, which was not investigated in previous studies. In this study, our deep learning based method can be used to assess the CACS accurately on the LM segment. Further study working on calcification distribution, especially at the segmental level, is expected.

This study has its limitations. Firstly, other cardiac and non-cardiac findings, including non-coronary artery cardiac calcification and aortic calcification, should also be reported according to the CAC-DRS [7]. Calcification at aortic root and annular may also affect the CACS quantification. In this study, our deep learning algorithm was designed and verified for only the CACS assessment. In the future study, a more comprehensive model should be developed for the consideration of non-coronary artery cardiac calcification and aortic calcification. Secondly, coronary artery origin anomalies patients were not enrolled in this study. Coronary artery origin anomalies are uncommon coronary artery congenital malformation in clinic practice. Accurate segmentation of coronary artery is essential for CACS assessment in coronary artery origin anomalies patients, which need abundant training data including different kinds of coronary artery origin anomalies. Thirdly, the patients in the testing dataset were significantly older than patients in the training dataset. The current study has evaluated the accuracy of calcification measurement on the same type of data (including blood vessels with and without calcification). Therefore, the impact of age differences could be mitigated. Lastly, our current study was a single-centre study using data acquired from a single CT machine. A further multi-centre and multi-scanner study should be carried out to verify the proposed automated framework.

In conclusion, the proposed fully automatic framework for CACS evaluation, including comprehensive calcification extent and distribution assessment, can provide accurate information and indicators to improve the cardiovascular risk discrimination and reclassification.

## Guarantor

The scientific guarantor of this publication is Lei Xu.

## Statistics and biometry

No complex statistical methods were necessary for this paper.

## Informed consent

Only if the study is on human subjects:

Written informed consent was waived by the Institutional Review Board.

## Ethical approval

Institutional Review Board approval was obtained.

## Study subjects or cohorts overlap

No study subjects or cohorts have been previously reported.

## Methodology

• retrospective
• diagnostic study
• performed at one institution

## CRediT authorship contribution statement

**Nan Zhang:** Validation, Formal analysis, Investigation, Writing - original draft, Writing - review & editing, Visualization. **Guang Yang:** Writing - review & editing. **Weiwei Zhang:** Software. **Wenjing Wang:** Formal analysis, Resources. **Zhen Zhou:** Formal analysis, Resources. **Heye Zhang:** Software. **Lei Xu:** Conceptualization, Supervision, Project administration, Funding acquisition. **Yundai Chen:** Conceptualization, Resources, Supervision, Project administration, Funding acquisition.

## Declaration of Competing Interest

The authors of this manuscript declare no relationships with any companies, whose products or services may be related to the subject matter of the article.

## Figures and Tables

**Figure:**
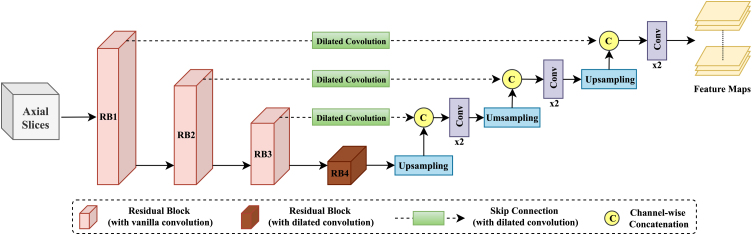


**Figure:**