# Supplementary material for: Fully automatic framework for comprehensive coronary artery calcium scores analysis on non-contrast cardiac-gated CT scan: Total and vessel-specific quantifications
Source: Eur J Radiol. 2021 Jan;134:109420. doi: 10.1016/j.ejrad.2020.109420 (PMC7814341; doi:10.1016/j.ejrad.2020.109420)
Supplement: Supplementary file 1 [file mmc1.docx]

**Supplementary material**

**Mathematical Formulation**

Our proposed MVSC formulates vessel-specific calcification analysis as a multi-task learning problem. The segmentation task ${\{y}_{\mathrm{Seg}}^{k}\}$ and the regression tasks ${\{y}_{\mathrm{AS}}^{k}, y_{\mathrm{VS}}^{k}{, y}_{\mathrm{MS}}^{k}\}$ are simultaneously learned for vessel-specific calcification analysis from the given CT scans ${\{X}^{k}\}$: *k = 1, · · ·, K* denotes the subject index. The objective of our MVSC is defined as,

$\begin{matrix} \min\\ W \end{matrix}\frac{1}{K}\sum_{k} \sum_{t} lt\left( f\left( X^{k},W \right), y_{t}^{k} \right)+\lambda\parallel W\parallel_{2}$ (1)

where $t\in\{Seg, AS, VS, MS\}$ denotes a specific objective task, $lt$ denotes the loss function of the task *t*, and$\parallel W\parallel_{2}$ denotes the L2 regularisation of the network parameters.

**Residual Dilated Network for Axial View Learning**

We model the *axial slices* by a residual dilated convolutional network (RDCN) module, which consists of a contracting and an expansive path (Figure S1). In the contracting path, widely used deep residual networks (ResNet) with four residual blocks is employed to encode the input axial slices. These residual blocks compose of 3, 4, 6, 3 residual modules, respectively. Each residual module has three successive convolution layers with a kernel size of 1 × 1, 3 × 3, and 1 × 1. Each convolutional layer is followed by a batch normalisation layer (BN) to normalise the output feature maps and a rectified linear unit (ReLU) to improve the sparsity. In addition, the last residual block replaces the standard convolution by the dilated convolution (dilation rate is set to 2) to make the effective receptive field of the output feature maps larger than the original input images. Between every two adjacent residual blocks, the max pooling operation is applied to reduce dimensionality while saving computational memory. In the expansive path, the up-sampling operation is used to recover the dimension of the feature maps, which is followed by concatenation with the corresponding level feature maps of the contracting path and two 3 × 3 convolutions with 16 kernels, respectively.

For the skip connection between the contracting path and the expansive path, our RDCN incorporates three successive dilated convolution layers to assemble the feature maps from the contracting path instead of directly propagating them to the expansive path that is loosely inspired by the global convolutional network. Each dilated convolution contains 16 kernels of 3 × 3 with a dilation rate of 2. The incorporated dilated convolutions can not only reduce the number of channels but can also enlarge the valid receptive field to yield an effective global semantic feature representation.

**Multi-View Shape Constraint for Multiview Learning**

Calcification lesions are usually small, irregular and span multiple axial slices; therefore, we have developed a multiview learning model with attention and shape constraint to improve calcification detection. For the *coronal and sagittal views*, we propose a DC-UNet architecture to model them and obtain complementary information about calcification lesions (Figure S2). The DC-UNet replaces the skip connections of the UNet with dilated convolutions to incorporate the advantages of UNet and dilated convolution. At the end of the DC-UNet, a sigmoid layer is used to normalise its output to the range of [0,1]. The predicted maps served as the attention mask are integrated with the RDCN, which can be considered as a trunk branch to form the attention model. This mask branch can enhance the effective features but suppress the noise ones that can eventually improve the discriminability of the RDCN. Our attention model can be formulated as,

${AM}_{mv}\left( x_{i,c} \right)=\left( 1+M_{cv}\left( x_{i,c} \right)+M_{sv}\left( x_{i,c} \right) \right)*F_{av}\left( x_{i,c} \right)$ (2)

where $i$ and $c$ indicate the indices of spatial positions and channels, respectively, and $x_{i,c}$ indicates the feature vector of the *i*-th spatial position, *c*-th channel. $M_{\mathrm{CV}}\left( x_{i,c} \right)$ and $M_{\mathrm{SV}}\left( x_{i,c} \right)$ denote the predicted maps from the coronal view (CV) and sagittal view (SV), respectively. $F_{\mathrm{AV}}\left( x_{i,c} \right)$ denotes the output of the RDCN of the axial view (AV). ${AM}_{\mathrm{MV}}\left( x_{i,c} \right)$ represents the features learned by the multiview (MV) attention model.

**Vessel-Specific Calcification Estimation**

With the output of our multiview attention model, a segmentation model and a regression model are incorporated to estimate the related calcification indices. In the segmentation model, high-resolution features from the previous down-sampling module are combined with the multiview attention features for precise vessel-specific calcification detection. Next, a convolution layer followed by a BN layer and a ReLU layer converts the number of channels into 4, and an up-sampling layer recovers the feature maps to the original image size. In the regression model, three fully connected layers of size 128 × 128 × 16, 64, 12 with a dropout layer and an addition operation along with the first dimension are applied to regress the calcium scores. For the loss function in formula (1), F1-scores are employed for the segmentation and regression tasks, respectively.

**Implementation details.**

We have used an Adam method to perform optimisation of the network with an initial learning rate of 0.002 (gradually decay to 0.0001). Dropout rate in regression model is set to 0.5. Regularisation coefficient of the loss function is set to 0.0001. After training is completed, the independent testing is directly executed on the learned training model. Our MVSC has been implemented using Tensorflow 1.8.0, and has been trained and tested on an NVidia Tesla P40 GPU (24GB GPU memory).

**Supplemental Figure Legends**

Figure S1: The network architecture of the proposed RDCN, which is used for the axial view. RDCN adopts the most popular classification model ResNet as the skeleton and improves ResNet by the dilated convolution. Followed the enhanced ResNet, RDCN adds an extended path to recover the size of feature maps. The ‘x2’ below Conv denotes two consecutive convolutions.

Figure S2: The network architecture of the proposed DC-UNet, which is used for the coronal and sagittal views. DC-UNet is developed based on the combination of U-net and dilated convolution. The numbers above feature maps represent their channels.
